# Changes in Plasma Metabolic Signature upon Acute and Chronic Morphine Administration in Morphine-Tolerant Mice

**DOI:** 10.3390/metabo13030434

**Published:** 2023-03-16

**Authors:** Naseer A. Kutchy, Amelia Palermo, Rong Ma, Zhong Li, Alexandria Ulanov, Shannon Callen, Gary Siuzdak, Sabita Roy, Shilpa Buch, Guoku Hu

**Affiliations:** 1 Department of Animal Sciences, Rutgers, The State University of New Jersey, New Brunswick, NJ 08901, USA; 2 Department of Pharmacology and Experimental Neuroscience, University of Nebraska Medical Center, Omaha, NE 68198, USAsbuch@unmc.edu (S.B.); guoku.hu@unmc.edu (G.H.); 3 Department of Molecular and Medical Pharmacology, David Geffen School of Medicine, University of California, Los Angeles, CA 90095, USA; 4 Department of Pharmacology, School of Basic Medicine, Tongji Medical College, Huazhong University of Science and Technology, Wuhan 430030, China; 5 Department of Biostatistics & Bioinformatics, Duke University School of Medicine, Durham, NC 27710, USA; 6 Roy J. Carver Biotechnology Center, University of Illinois, Urbana, IL 61801, USA; 7 Center for Metabolomics and Mass Spectrometry, Scripps Research Institute, La Jolla, CA 92037, USA; 8 Department of Surgery, University of Miami, Miami, FL 33136, USA

**Keywords:** morphine, opioids, tolerance, metabolism, plasma, metabolomics, GC-MS

## Abstract

Morphine administration causes system-level metabolic changes. Here, we show that morphine-tolerant mice exhibited distinct plasma metabolic signatures upon acute and chronic administration. We utilized a mouse model of morphine tolerance by exposing mice to increasing doses of the drug over 4 days. We collected plasma samples from mice undergoing acute or chronic morphine or saline injections and analyzed them using targeted GC–MS-based metabolomics to profile approximately 80 metabolites involved in the central carbon, amino acid, nucleotide, and lipid metabolism. Our findings reveal distinct alterations in plasma metabolite concentrations in response to acute or chronic morphine intake, and these changes were linked to the development of tolerance to morphine’s analgesic effects. We identified several metabolites that had been differentially affected by acute versus chronic morphine use, suggesting that metabolic changes may be mitigated by prolonged exposure to the drug. Morphine-tolerant mice showed a restoration of amino acid and glycolytic metabolites. Additionally, we conducted reconstructed metabolic network analysis on the first 30 VIP-ranked metabolites from the PLSDA of the saline, acute, and morphine-tolerant mice groups, which uncovered four interaction networks involving the amino acid metabolism, the TCA cycle, the glutamine-phenylalanine-tyrosine pathway, and glycolysis. These pathways were responsible for the metabolic differences observed following distinct morphine administration regimens. Overall, this study provides a valuable resource for future investigations into the role of metabolites in morphine-induced analgesia and associated effects following acute or chronic use in mice.

## 1. Introduction

Morphine is widely recognized as an effective analgesic for managing moderate-to-severe pain during surgeries and in several debilitating diseases such as cancer [1,2,3]. After intake, morphine undergoes hepatic metabolism via glucuronidation to morphine-6-glucuronide (M6G) and morphine-3-glucuronide (M3G). M6G is biologically active and exhibits a stronger analgesic effect than that of its parent compound and the M3G isomer owing to the higher affinity of M6G for the μ-opioid receptor [4,5,6,7,8,9,10]. In addition to its analgesic effect, prolonged morphine intake also results in other undesired effects such as addiction, dependence, and tolerance, which necessitate its cautious use in clinical settings, particularly for patients with a history of chronic morphine use.

Metabolomics, a comprehensive measurement of metabolites in biological samples, was applied to exploring metabolic changes associated with the use of morphine and other addictive substances [11,12,13,14,15,16,17,18]. Previous investigations reported unique changes in plasmatic metabolic profiles following morphine intake. For example, Zaitsu et al. demonstrated that prolonged intravenous morphine use resulted in modified plasma metabolic patterns in a rat model of addiction, and plasma metabolites could serve as predictors of addiction [19]. Another study reported changes in plasma metabolites in rats during the euphoria, tolerance, and withdrawal phases of morphine use after 7 days of acclimatization [20]. Caspani et al. provided a comprehensive review of metabolomic studies of the metabolic signature underlying opioids and morphine addiction [21]. More recently, plasma metabolic profiles were proposed as potential predictors of patient response to opioids [22].

Despite this, morphine-mediated changes in metabolites could also influence tolerance development, but there is paucity of information about the impact of morphine administration on plasma metabolites following chronic or acute use in the context of morphine tolerance [23,24]. Various rodent models of morphine tolerance were developed, such as subcutaneous or intraperitoneal injection of morphine for 4–14 days [23,24,25,26,27,28,29,30,31,32] and morphine pellet implantation [25,33,34,35]. C57/6B mice were subjected to a well-established tolerance regimen in which mice were subcutaneously administered either saline or morphine for 4 days. The development of morphine tolerance was evaluated via the cumulative dose–response data from before (Day 1) and after (Day 5) chronic morphine administration. Using this model, the current study investigates changes in the plasma metabolite signature of mice administered with acute or chronic morphine. The findings from this study may identify metabolic targets for further investigation into their role in morphine tolerance.

## 2. Material and Methods

### 2.1. Study Design

**Figure 1:**
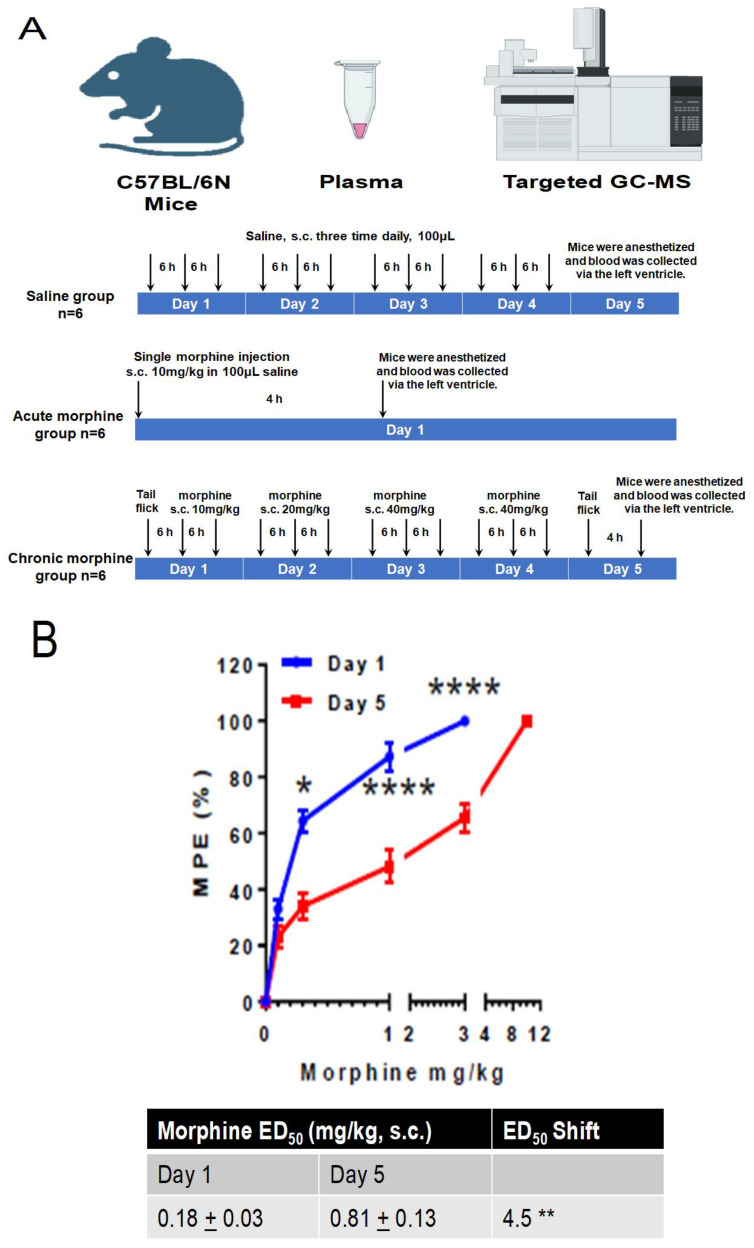
Schematization of study design and morphine-tolerance mice model development. (**A**) Schematic depicting morphine dosing and behavioral testing paradigm for morphine-induced tolerance experiments. (**B**) Dose–response curve of morphine in C57BL/6N mice. Cumulative dose–response studies were performed before (Day 1) and after (Day 5) morphine treatment. Error bars represent SEM from 6 mice. * *p*, 0.05, ** *p*, 0.001 and **** *p*, 0.0001 for an unpaired Student’s *t* test.

In the current study, we included three groups of mice: a control group that received saline injections, an acute group that was exposed to subcutaneous morphine administration for 4 h, and a chronic group that received the drug until the development of tolerance. Plasma samples were collected from the mice in each group, as shown in Figure 1. Targeted metabolomics using gas chromatography–mass spectrometry (GC–MS) was employed to analyze the plasma metabolites of the mice. The resulting data were normalized, and principal component analysis (PCA) and partial least-squares discriminant analysis (PLSDA) were performed using MetaboAnalyst 5.0 and Rscript chemometrics.

### 2.2. Animals

A total of 18 male C57BL/6N wild-type mice (6–8 weeks) were purchased from Charles River Laboratories, Inc. (Wilmington, MA, USA) and were kept in a controlled environment at the animal facility of the University of Nebraska Medical Center (UNMC) with ad libitum access to food and water, a 12 h light/dark cycle (lights on at 07:00 a.m.), and maintained at specific temperature and humidity levels (3–5 animals per cage). The mice were grouped into three categories: saline, acute morphine, and chronic morphine groups. The saline group (*n* = 6) received a subcutaneous injection of saline (100 μL) three times per day for four consecutive days. On the fifth day, their plasma was collected, and they were euthanized. The acute morphine group (*n* = 6) received a single subcutaneous injection of morphine (40 mg/kg, dissolved in 100 μL saline), and their plasma was collected four hours later before being sacrificed. For the chronic morphine group (*n* = 6), a cumulative dose–response assay was conducted on the first day with morphine dissolved in saline, and the mice were injected twice (s.c., 10 mg/kg) within a 6 h interval. On the second day, they received three injections (s.c., 20 mg/kg) within the same interval, and on Days 3 and 4, they received three injections (s.c., 40 mg/kg) at the same interval to induce and maintain tolerance levels [36]. On Day 5, cumulative morphine dose–response assays were conducted again. Four hours after the cumulative dose–response assay, their plasma was collected, and they were euthanized. The experimental protocol involving the animals was examined and approved by the Institutional Animal Care and Use Committee (IACUC) at UNMC.

### 2.3. Morphine Analgesia and Tolerance

An LE7106 analgesia meter (Panlab Harvard, MA, USA, focus intensity: 30) was used for tail-flick assays to evaluate the analgesic effect of morphine in the mice. To ensure reliable results and minimize stress, the mice were handled and acclimated to entering the restrainer for 5 min daily for 7 days prior to testing. Mice were also acclimated to the experimental room for at least 1 h to adjust to the environment and reduce stress. Before each test session, all mice were habituated to the tail-flick device for 2 min. After measuring baseline latency, the mice were injected with an initial dose of morphine at 0.1 mg/kg body weight, followed by increasing doses of 0.3, 1, 3, and 10 mg/kg. Tail-flick tests were conducted 30 min after each dose, with the next dose of morphine administered immediately thereafter. Tail-flick latency was measured before and 1 day after morphine administration (as depicted in Figure 1). A cut-off time of 10 s was established for the tail flick to prevent harm to the mice. The antinociceptive response was calculated as a percentage of the maximal possible effect (MPE), where MPE % = (test latency − baseline latency) × 100/(cutoff latency − baseline latency). Following the experiments, the mice were anesthetized, and blood was collected through the left ventricle using a 23–25 gauge needle.

### 2.4. Metabolite Profiling

Sample preparation: 50 μL of plasma was mixed with 0.5 mL extraction mixture (methanol/water/isopropanol, 3/2/3 *v*/*v*/*v*). The supernatant was evaporated and derivatized with 80 μL methoxyamine hydrochloride (40 mg/mL) for 60 min at 50 °C and with 80 μL *N*-methyl-*N*-(trimethylsilyl)trifluoroacetamide (MSTFA) at 70 °C for 120 min, then 2 h incubation at room temperature. We added 20 μL of the internal standard (hentriacontanoic acid, 1 mg/mL) to each sample prior to derivatization. 

Instrument analysis: metabolite profiling was performed using a GC–MS system (Agilent Inc, Santa Clara, CA, USA) consisting of an Agilent 7890 gas chromatographer, an Agilent 5975 MSD, and an HP 7683B autosampler. Gas chromatography was performed on a ZB-5MS (60 m × 0.32 mm I.D. and 0.25 Um film thickness) capillary column (Phenomenex, Torrance, CA, USA). The inlet and MS interface temperatures were 250 °C, and the ion source temperature was adjusted to 230 °C. An aliquot of 1 μL was injected with the split ratio of 10:1. The helium carrier gas was kept at a constant flow rate of 2 mL/min. The temperature program was: 5 min of isothermal heating at 70 °C, followed by an oven temperature increase of 5 °C per minute to 310 °C and a final 10 min at 310 °C. The mass spectrometer was operated in positive electron impact mode (EI) at 69.9 eV ionization energy at a scan range of *m*/*z* 30–800.

Metabolite data analysis: the spectra of all chromatogram peaks were evaluated using AMDIS 2.71 (NIST, Gaithersburg, MD, USA) software using a custom-built database (460 unique metabolites). All known artificial peaks were identified and removed prior to data mining. To allow for a comparison between the samples, all data were normalized to the internal standard in each chromatogram and the sample weight. Instrument variability was within the standard acceptance limit (5%).

### 2.5. Data Analysis

Metabolite abundances were examined with univariate statistical analysis in R Studio (*t*-test significance analysis, two-tailed distribution with heteroscedastic variance). Metabolite abundances were considered significantly different for *p* < 0.05 and absolute fold change > +1.5. Prior multivariate analysis (principal component analysis (PCA), partial least-squares discriminant analysis (PLSDA)) metabolite abundance was normalized as follows: log-transformed and autoscaled (i.e., mean-centered and divided by the standard deviation of each variable) to normal distribution. Metabolite abundances with fewer than three replicates were excluded from analysis. The PCA and PLSDA of normalized metabolite abundance were performed using MetaboAnalyst 5.0 with the Rscript chemometrics, R, [37]. Sample hierarchical clustering was performed using the hclust function in the stat. R package (Ward clustering with Euclidean distance measure). Metabolite network analysis was performed with MetScape 3 in Cytoscape [38,39].

## 3. Results

### 3.1. Metabolite Changes in Plasma Postacute or Chronic Morphine Intake in Tolerant Mice

We first developed a morphine-tolerant mouse model with the subcutaneous injection of a well-established tolerance regimen (doses of morphine from 10 to 40 mg/kg over 4 days—Figure 1A). The development of morphine tolerance was determined via cumulative dose–response data starting at the baseline (Day 1) and post (Day 5) chronic morphine administration (Figure 1A). Chronic morphine administration significantly shifted the tail-flick response curve to higher morphine dosage, with increased ED50 values (i.e., morphine dose effective in 50% of the animals) (Figure 1B,C). The same mouse model was also exposed to acute morphine intake (single i.v. 10 mg/kg dose). Mice injected with saline were used as a control.

**Figure 2:**
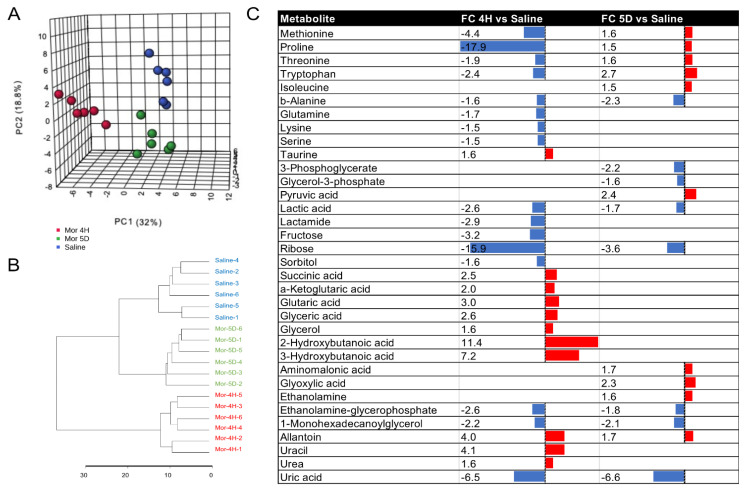
Acute or chronic morphine intake is associated with distinct plasma metabolic signatures. (**A**) Principal component analysis of metabolic profiles detected with GC–MS in mouse plasma after acute, 4 h (Mor 4H), chronic, 5 days (Mor 5D), or after saline (Saline) injection of morphine; (**B**) hierarchical clustering of metabolic signatures in distinct mice groups; (**C**) univariate analysis of significant metabolic changes occurring in mouse plasma after acute (4H vs. Saline) or chronic (5D vs. Saline) intake of morphine in tolerant mice. Metabolites were considered significant for *p*-values < 0.05 in significance *t*-testing (2-tailed, heteroscedastic), and absolute fold change (FC) > 1.5; red bar: upregulation, blue bar: downregulation.

Next, we collected plasma samples from mice after 4 h (4H group, acute) and after 5 days (5D group, chronic) of morphine intake. In total, 80 metabolites in central carbon, amino acid, and lipid metabolism were measured with targeted GC–MS analysis (Supplementary Table S1). Principal component analysis (PCA) of the metabolites measured in at least 3 samples showed distinct metabolic signatures among the control, 4H, and 5D groups, with principal component (PC) 1 explaining 32%, and PC 2 explaining 18.8% of the total variance in the data set (Figure 2A). Metabolic profiles from samples collected after chronic morphine use were positioned between the saline and 4 h groups along PC1, suggesting that metabolic changes caused by acute intake are mitigated after 5 days of escalated chronic administration. The hierarchical clustering of samples according to computed Euclidean distances across metabolic profiles corroborates this observation (Figure 2B).

Univariate statistical analysis of metabolite abundance in the 4H vs. saline and 5D vs. saline groups demonstrated that a total of 27 and 18 metabolites had changed after acute or chronic morphine intake, respectively (Figure 2C).

Acute morphine administration led to increased TCA cycle intermediates (succinic acid and a-ketoglutaric acid), taurine, glutaric acid, lipid metabolites (2- and 3-hydroxybutanoic acid, glycerol and glyceric acid), and uracil. Both essential and nonessential amino acids (methionine, proline, threonine, tryptophan, isoleucine, alanine, glutamine, lysine, serine), and glycolytic metabolites (lactic acid, lactamine, fructose, ribose and sorbitol) were decreased following acute intake.

Following chronic exposure to the escalating morphine regimen, amino acid levels were rescued or significantly increased (methionine, proline, threonine, isoleucine, glutamine, lysine, serine) with the exception of alanine. Changes in the TCA intermediates and hydroxybutanoic and glyceric acids were also reversed. Our data also show increased pyruvate and decreased glycerol-3-phosphate in glycolytic metabolism in the chronic morphine regimen. This was accompanied by decreased changes in ribose and uracil levels. Alanine, lactic acid, ethanolamine glycerophosphate, 1-monohexadecanoylclycerol, allantoin, and uric acid showed consistent changes across acute and chronic intake.

### 3.2. Pathways and Networks of Plasma Metabolites in Acute versus Chronic Intake

**Figure 3:**
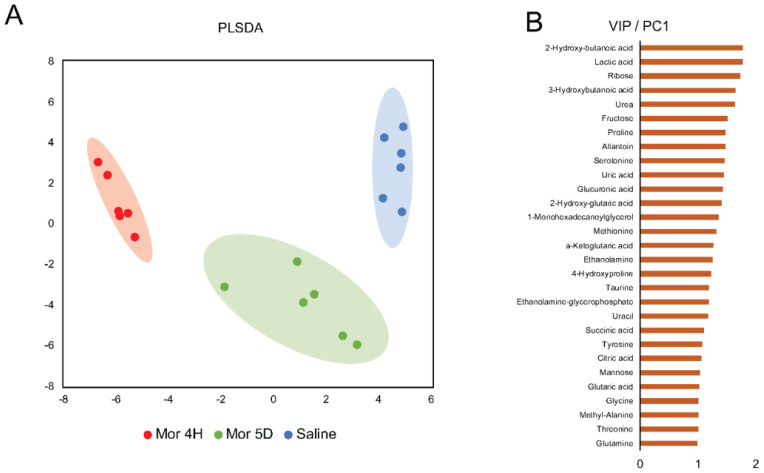

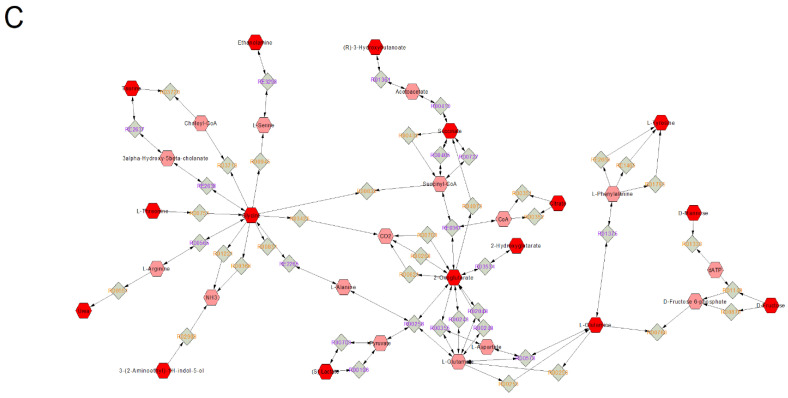
Metabolic network analysis of the first 30 metabolites, ranked according to the VIP score on PLSDA PC1, responsible for changes in metabolite plasma concentrations in the 4H, 5D, and Saline groups. (**A**) PLSDA dimensionality reduction of metabolic profiles to PC1 and PC2; (**B**) VIP scores as projected on PC1 for first 30-ranked metabolites; (**C**) metabolic network reconstruction analysis showing four main networks centered on amino acid, TCA cycle, and glycolytic metabolism. Red nodes represent metabolites among the first 30-ranked features according to VIP scores, edges represent metabolic enzymes responsible for metabolite biotransformations, and orange nodes represent metabolites not reported among the first 30 ranked VIP ones but involved in their biotransformations/metabolism.

To establish the underlying pathways and metabolic networks that determine distinct metabolic signatures observed following acute vs. chronic morphine intake, we performed partial least-squares discriminant analysis (PLSDA) of normalized abundances from measured metabolites. This supervised multivariate modeling approach maximizes the variance explained by each principal component to emphasize metabolic features responsible for group separation (Figure 3A). Our results show that PC1 explained the variation among the saline, 4H, and 5D groups. Next, we selected the first 30 metabolites ranked according to their variable importance in projection (VIP) score computed on PC1 (Figure 3B). Among these, 2- and 3- hydroxybutanoic acid, lactic acid, ribose, and urea.

Next, we used the first 30 VIP-ranked metabolites to reconstruct a network of metabolite-pathway interactions using MetScape 3 in Cytoscape. This approach allowed for the visualization of metabolite network and enzymes associated with metabolic variations observed in 4H, 5D, and saline groups. In particular, MetScape leverages Kyoto Encyclopedia of Genes and Genomes (KEGG) metabolite IDs as compound identifiers and contextualizes them in the relevant metabolic reactions in mouse-specific metabolic pathways [39]. We filtered the obtained network for only edges connecting, directly or through further metabolic reactions, at least two of the significant metabolites shortlisted according to their VIP score. This analysis unveiled four main interaction networks centered on amino acid metabolism: the TCA cycle, glutamine-phenylalanine-tyrosine metabolism, and glycolysis (Figure 3C), thus suggesting that adaptations in these pathways arising over the prolonged (chronic) use of morphine are responsible for the metabolic differences observed under distinct drug administration regimens.

## 4. Discussion

In this study. we applied a mouse model of morphine tolerance upon drug escalation (from 10 to 40 mg/kg over 4 days) and employed the model for establishing differences in plasma metabolic signatures after acute (4 h) or chronic (5 days, tolerance) morphine s.c. administration. Saline-injected mice were used as controls.

Our data demonstrate that plasma metabolic signatures in morphine-tolerant mice were distinct from those induced by acute intake. Animals receiving a single dose of the opioid showed increased TCA cycle intermediates, taurine, glutaric acid, and uracil, while amino acid, ribose, and lactate levels were decreased. In contrast, the chronic administration of morphine reversed changes in amino acids (except alanine and taurine), TCA cycle intermediates, and hydroxybutanoic and glyceric acids. This was accompanied by increased pyruvate and decreased glycerol-3-phosphate, and reduced changes in uracil and ribose. We detected consistent changes across chronic and acute intake for alanine, lactic acid, ethanolamine glycerophosphate, 1-monohexadecanoylglycerol, allantoin, and uric acid.

In a previous investigation of plasma for 41 metabolites in rats, Liu et al. reported reduced overall metabolic changes with increased 4-hydroxybutanoic acid during morphine-induced tolerance compared with animals sampled 30 min post morphine intake (euphoria phase) [20]. Consistent with this study, our data show mitigated metabolic changes over prolonged opioid exposure to tolerance. By establishing metabolic changes in plasma after acute and chronic morphine intake in a well-established mouse model of tolerance, our investigation provides evidence for the association between tolerance and plasma metabolite signatures by measuring > 80 metabolic features.

Further studies will be focused on defining metabolic changes induced by morphine in the brain. In particular, rat addiction to morphine was associated with disrupted brain energy metabolism in TCA and glycolytic pathways. That study reported decreased plasma tryptophan in the plasma of addicted rats, a consistent finding with our observations after acute intake while contrasting with our data from tolerant mice [19]. Furthermore, extracellular vesicles (EVs) are natural carriers of metabolites that play a vital role in various cellular signaling pathways in the context of drug abuse [23,40,41,42,43]. The potential role of EV-associated metabolites in morphine tolerance warrants further investigation.

Morphine intake also causes increased g-amminobutyric acid (GABA) in the nucleus accumbens, prefrontal cortex, and striatum in the brain due to increased conversion of glutamine into glutamate via increased glutamic acid decarboxylase in rats after acute exposure. This may be the basis for the decreased glutamine plasma levels observed in our study after acute intake. Further changes in brain levels of succinate and a-ketoglutaric acid in the TCA were reported [44,45].

Increased taurine in the hippocampus, nucleus accumbens, and striatum was described in morphine-dependent rats and is in line with our observations in plasma in both acute and tolerant mice [46]. Similarly, Meng et al. showed increased proline in the brains of morphine-induced conditioned place preference mouse model, consistent with our findings [45].

Morphine-induced changes in brain metabolism, however, cannot solely account for metabolic changes observed in plasma, since the effect of morphine on the metabolic homeostasis of other tissues and organs contributes to overall variations. For instance, morphine intake increases the glycolytic flux and lactate formation with reduced pyruvate utilization in TCA metabolism in the brain [46,47]. However, our data show overall reduced lactate levels in plasma after both acute and chronic intake. Moreover, our study revealed that chronic morphine administration resulted in a 1.3-fold rise in plasma glucose levels compared to the saline group mice, while acute morphine administration had no significant impact on plasma glucose levels. These results are consistent with previous reports and indicate that the chronic use of morphine could aggravate diabetes, dyslipidemia, and hypertension [48,49,50].

The administration of naloxone, a m-opioid receptor antagonist that counteracts morphine’s effects, showed increased lactic acid in plasma after 5 days of oral morphine administration in rats [20]. This finding suggests that the decreased lactic acid detected in our data set following both acute and chronic intake could be rescued by antagonizing morphine pharmacodynamic activity.

Next, we input the first 30 VIP-ranked metabolites from the PLSDA of the saline, 4H, and 5D mice groups to reconstruct the network of metabolite and biotransformations underlying the observed differences in group clustering. This analysis revealed four interaction networks centered on amino acid metabolism: the TCA cycle, the glutamine-phenylalanine-tyrosine pathway, and glycolysis.

## 5. Conclusions

Taken together, our data demonstrate that metabolic profiles collected from morphine-tolerant mice vs. profiles obtained after acute intake were distinct. In particular, metabolic changes in morphine-tolerant mice were mitigated, raising the question of whether changes in the plasma metabolites following acute morphine intake were contributing to morphine analgesic effects and whether they could serve as tolerance indicators. The role of metabolic changes in morphine-mediated pathologies [43,51,52,53,54,55] demands additional studies. Our findings warrant further investigation in other morphine tolerance models and humans. Overall, this study constitutes a valuable resource for future investigations aimed at defining the role of metabolites in morphine-induced analgesia and associated effects after acute or chronic (mice-tolerant) intake.

## Data Availability

All the data in this study are contained within the manuscript.

## Supplementary Materials

The following supporting information can be downloaded at: https://www.mdpi.com/article/10.3390/metabo13030434/s1, Table S1: 80 metabolites.
